# Supplementary figures and images for: Autologous micro-fragmented adipose tissue in the treatment of atherosclerosis patients with knee osteoarthritis in geriatric population: A systematic review and meta-analysis
Source: PLoS One. 2023 Aug 31;18(8):e0289610. doi: 10.1371/journal.pone.0289610 (PMC10470951; doi:10.1371/journal.pone.0289610)

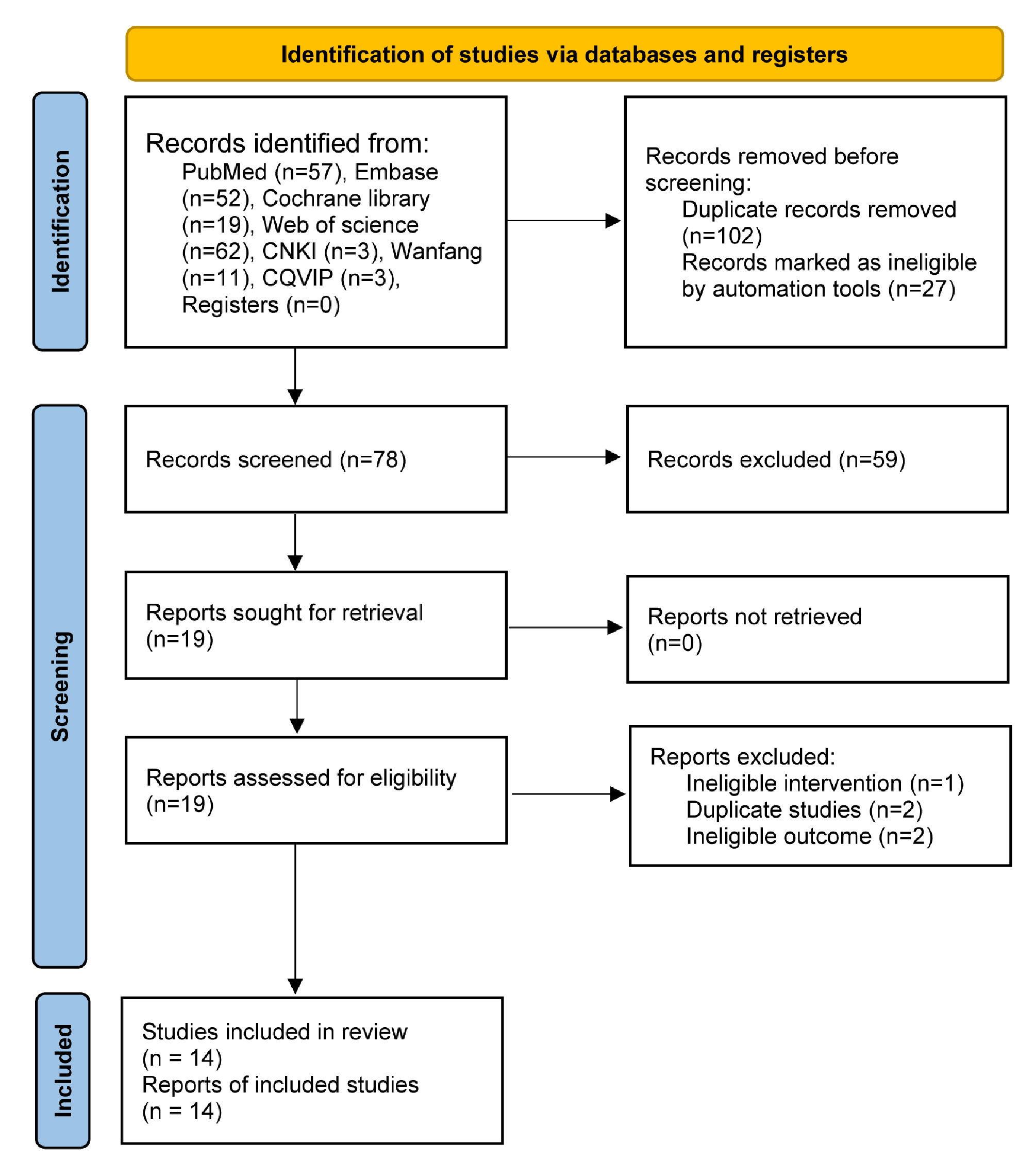

Supplement: S1 Fig — CNKI, China National Knowledge Infrastructure. (TIF) [file pone.0289610.s002.tif]

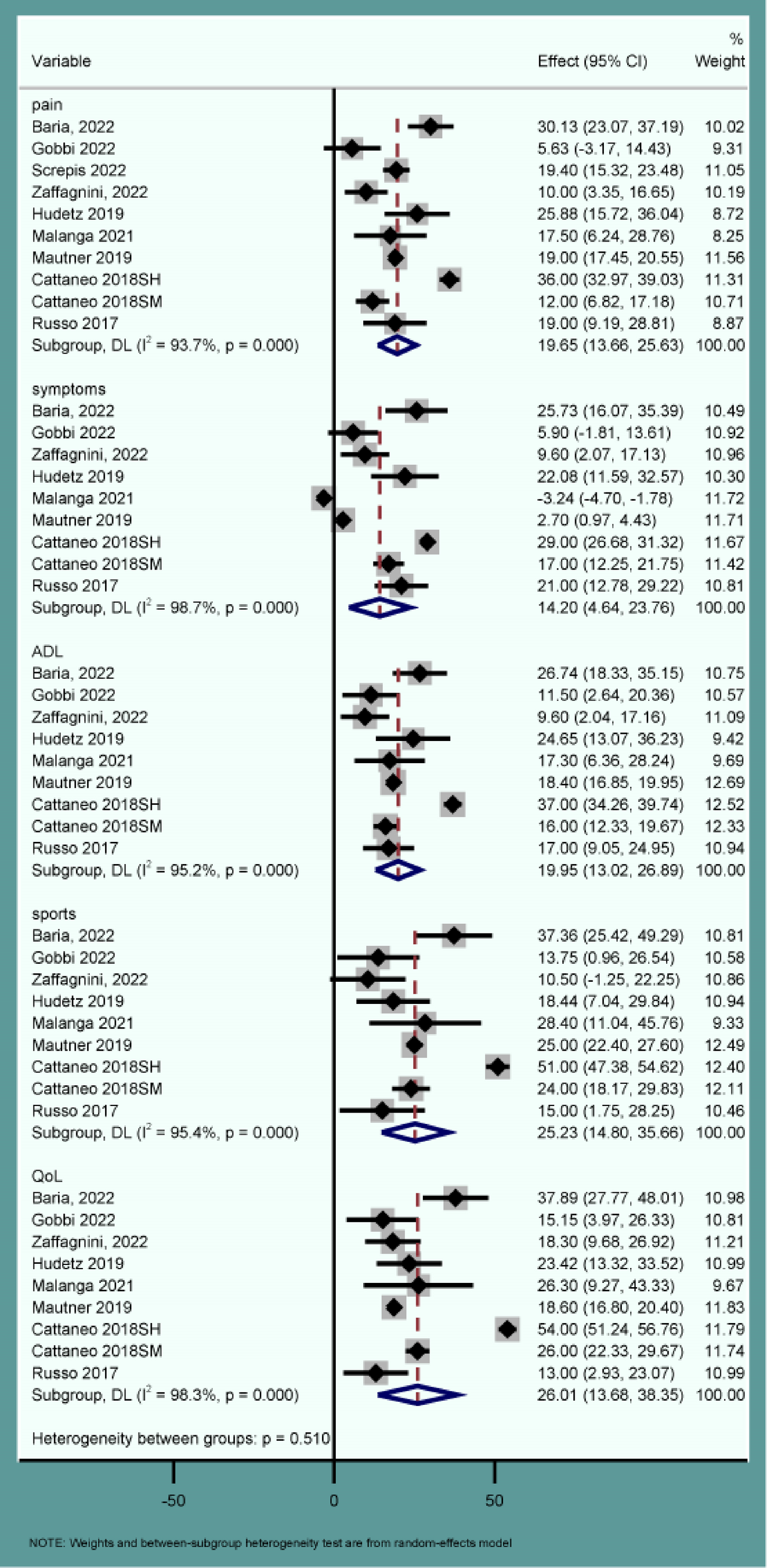

Supplement: S2 Fig — KOOS, Knee Injury and Osteoarthritis Outcome Score; ADL, activities of daily living; QoL, quality of life; CI, confidence interval. (TIF) [file pone.0289610.s003.tif]

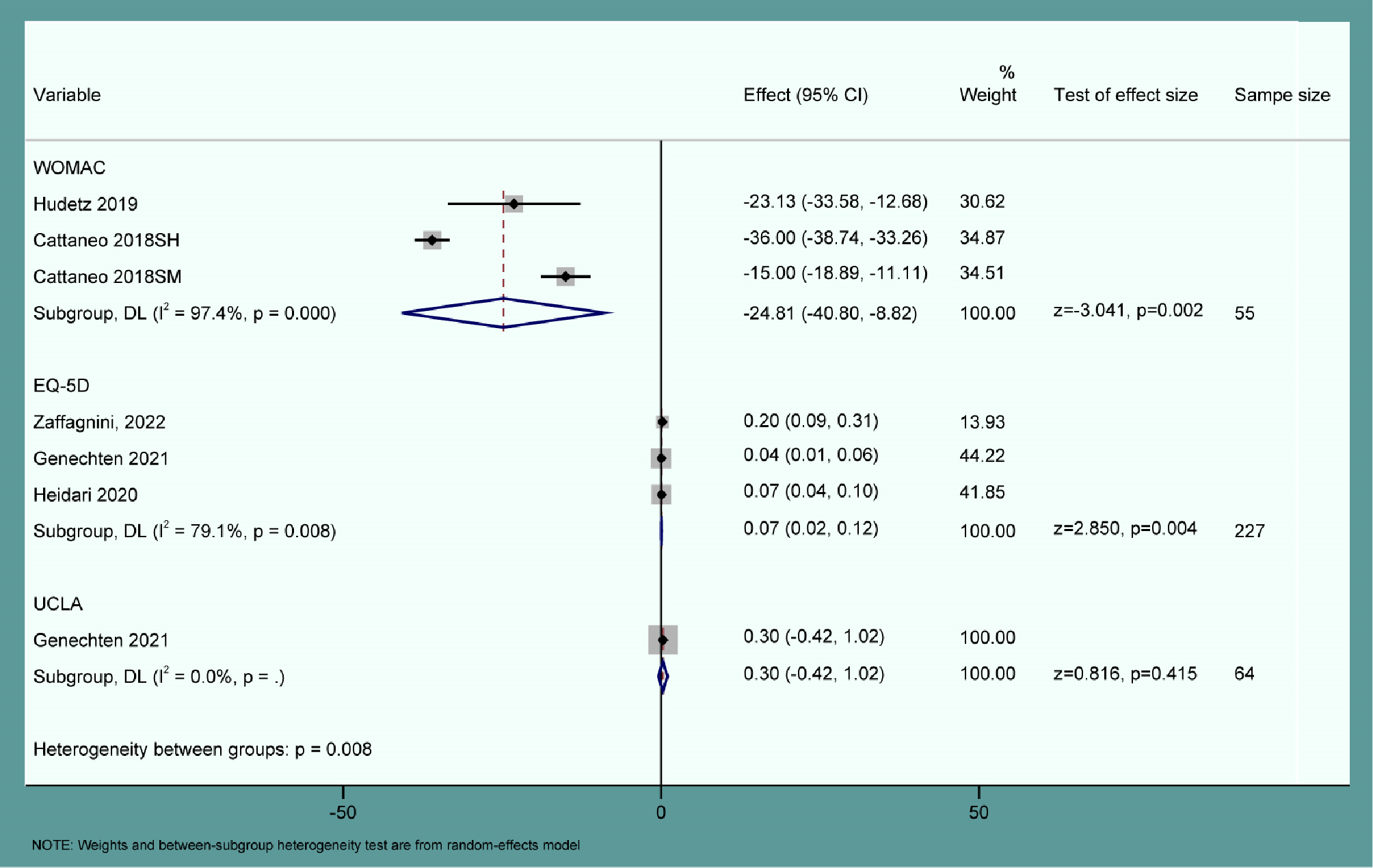

Supplement: S3 Fig — WOMAC, the Western Ontario and McMaster Universities Osteoarthritis Index; EQ-5D, European Quality of Life Five Dimension Five Level; UCLA, University of California in Los Angeles (UCLA); CI, confidence interval; DL, Dersimonian-Larid. (TIF) [file pone.0289610.s004.tif]

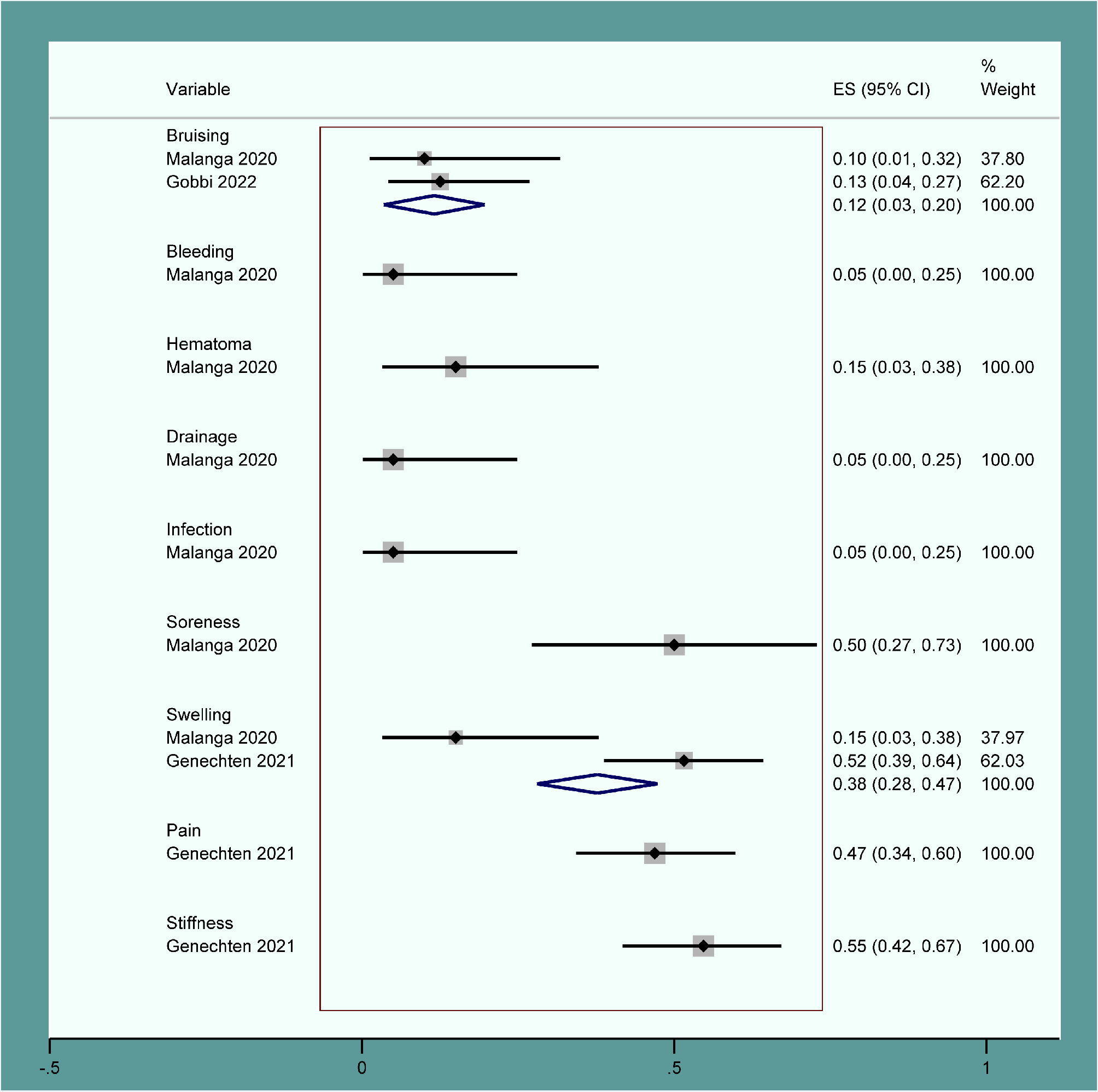

Supplement: S4 Fig — ES, effect size; CI, confidence interval. (TIF) [file pone.0289610.s005.tif]

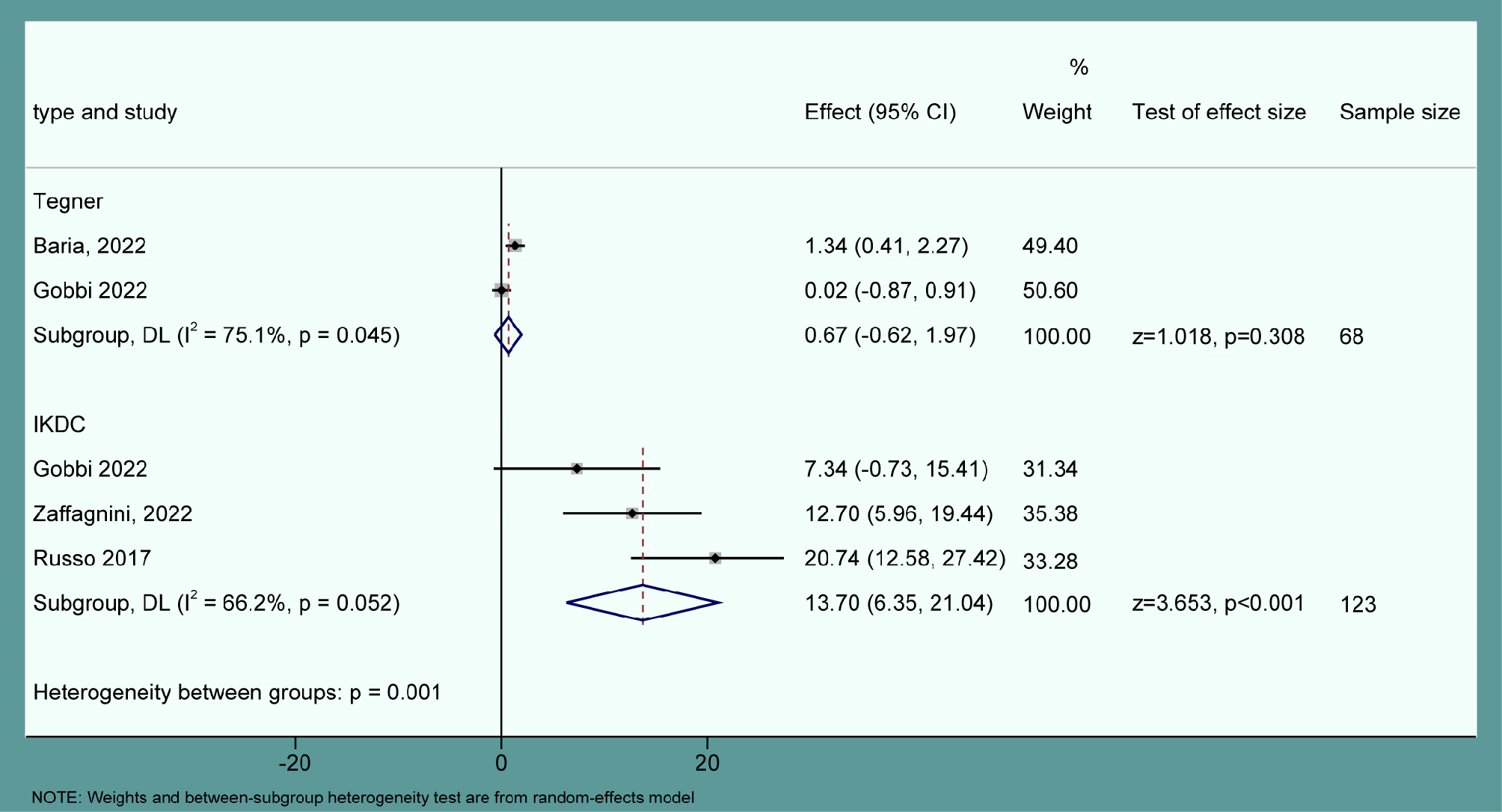

Supplement: S5 Fig — (TIF) [file pone.0289610.s006.tif]

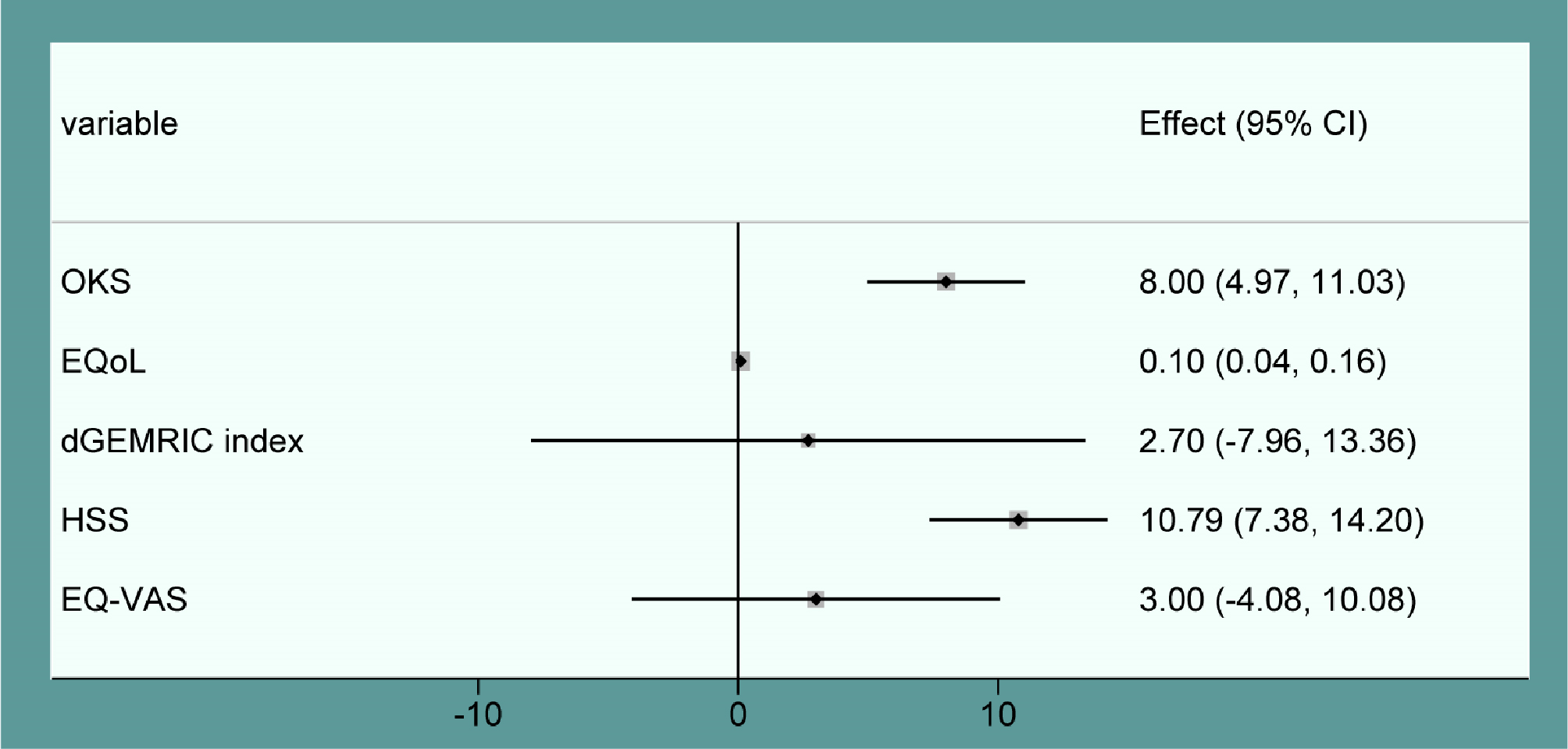

Supplement: S6 Fig — OKS, Oxford Knee Score; HSS, Hospital for Special Surgery Knee Score; EQoL, Emory Quality of Life; dGEMRIC, delayed gadolinium-enhanced magnetic resonance imaging of cartilage; CI, confidence interval; DL, Dersimonian-Larid. (TIF) [file pone.0289610.s007.tif]
