# Supplementary material for: Autologous micro-fragmented adipose tissue in the treatment of atherosclerosis patients with knee osteoarthritis in geriatric population: A systematic review and meta-analysis
Source: PLoS One. 2023 Aug 31;18(8):e0289610. doi: 10.1371/journal.pone.0289610 (PMC10470951; doi:10.1371/journal.pone.0289610)
Supplement: S1 Table — (DOCX) [file pone.0289610.s008.docx]

**Supplementary Table 1.** The detailed information of search strategies of target databases.

*Search strategy of PubMed*

| **#No** | **Search strategy** | **Results** |
| --- | --- | --- |
| **#1** | "Osteoarthritis"[Mesh] | 74131 |
| **#2** | (Osteoarthritides) OR (Osteoarthrosis) OR (Osteoarthroses) OR (Arthritis, Degenerative) OR (Arthritides, Degenerative) OR (Degenerative Arthritides) OR (Degenerative Arthritis) OR (Arthrosis) OR (Arthroses) OR (Osteoarthrosis Deformans) | 493775 |
| **#3** | **#1 OR #2** | 493775 |
| **#4** | "Osteoarthritis, Knee"[Mesh] | 25593 |
| **#5** | (Knee Osteoarthritides) OR (Knee Osteoarthritis) OR (Osteoarthritis of Knee) OR (Osteoarthritis of the Knee) | 46135 |
| **#6** | **#4 OR #5** | 46135 |
| **#7** | **#3 OR #6** | 493775 |
| **#8** | (Autologous Microfragmented Fat Tissue) OR (Microfragmented Adipose Tissue) OR (Micro-fragmented adipose tissue) OR (autologous micro-fragmented adipose tissue) OR (autologous microfragmented adipose tissue) OR (micro-fragmented lipoaspirate) | 104 |
| **#9** | #7 AND #8 | **57** |

*Search strategy of Embase*

| #No | Search strategy | Results |
| --- | --- | --- |
| #1 | 'osteoarthritis'/exp | 155216 |
| #2 | 'arthritis, degenerative' OR 'arthritis, noninflammatory' OR 'arthrosis' OR 'degenerative arthritis' OR 'degenerative joint disease' OR 'noninflammatory arthritis' OR 'osteo-arthritis' OR 'osteo-arthrosis' OR 'osteoarthrosis' OR 'primary osteoarthritis' OR 'rheumatoid arthrosis' | 20135 |
| #3 | 'knee osteoarthritis'/exp | 42048 |
| #4 | 'arthrosis, knee' OR 'femorotibial arthrosis' OR 'gonarthrosis' OR 'knee arthrosis' OR 'knee joint arthrosis' OR 'knee joint osteoarthritis' OR 'knee osteo-arthritis' OR 'knee osteo-arthrosis' OR 'knee osteoarthrosis' OR 'osteoarthritis, knee' OR 'osteoarthrosis, knee' | 3450 |
| #5 | #1 OR #2 | 162140 |
| #6 | #3 OR #4 | 42817 |
| #7 | #5 OR #6 | 162467 |
| #8 | 'microfragmented adipose tissue'/exp | 12 |
| #9 | ‘Autologous Microfragmented Fat Tissue’ OR ‘Microfragmented Adipose Tissue’ OR ‘Micro-fragmented adipose tissue’ OR ‘autologous micro-fragmented adipose tissue’ OR ‘autologous microfragmented adipose tissue’ OR ‘micro-fragmented lipoaspirate’ | 106 |
| #10 | #8 OR #9 | 106 |
| #11 | #7 AND #10 | **52** |

*Search strategy of Cochrane library*

| #No | Search strategy | Results |
| --- | --- | --- |
| #1 | MeSH descriptor: [Osteoarthritis] explode all trees | 8667 |
| #2 | (Osteoarthritides) OR (Osteoarthrosis) OR (Osteoarthroses) OR (Arthritis, Degenerative) OR (Arthritides, Degenerative) OR (Degenerative Arthritides) OR (Degenerative Arthritis) OR (Arthrosis) OR (Arthroses) OR (Osteoarthrosis Deformans) | 1681 |
| #3 | #1 OR #2 | 9936 |
| #4 | MeSH descriptor: [Osteoarthritis, Knee] explode all trees | 5235 |
| #5 | (Knee Osteoarthritides) OR (Knee Osteoarthritis) OR (Osteoarthritis of Knee) OR (Osteoarthritis of the Knee) | 15173 |
| #6 | #4 OR #5 | 15173 |
| #7 | #3 OR #6 | 18227 |
| #8 | (Autologous Microfragmented Fat Tissue) OR (Microfragmented Adipose Tissue) OR (Micro-fragmented adipose tissue) OR (autologous micro-fragmented adipose tissue) OR (autologous microfragmented adipose tissue) OR (micro-fragmented lipoaspirate) | 32 |
| #9 | #7 AND #8 | 19 |

*Search strategy of Web of Science (WoS)*

| #No | Search strategy | Results |
| --- | --- | --- |
| #1 | TS=(Osteoarthritis OR Osteoarthritis, Knee) | 171056 |
| #2 | TI=(Osteoarthritides OR Osteoarthrosis OR Osteoarthroses OR Arthritis, Degenerative OR Arthritides, Degenerative OR Degenerative Arthritides OR Degenerative Arthritis OR Arthrosis OR Arthroses OR Osteoarthrosis Deformans OR Knee Osteoarthritides OR Knee Osteoarthritis OR Osteoarthritis of Knee OR Osteoarthritis of the Knee) | 28046 |
| #3 | AB=(Osteoarthritides OR Osteoarthrosis OR Osteoarthroses OR Arthritis, Degenerative OR Arthritides, Degenerative OR Degenerative Arthritides OR Degenerative Arthritis OR Arthrosis OR Arthroses OR Osteoarthrosis Deformans OR Knee Osteoarthritides OR Knee Osteoarthritis OR Osteoarthritis of Knee OR Osteoarthritis of the Knee) | 48849 |
| #4 | #1 OR #2 OR #3 | 181484 |
| #5 | TS=microfragmented adipose tissue | 70 |
| #6 | TI=(Autologous Microfragmented Fat Tissue OR Microfragmented Adipose Tissue OR Micro-fragmented adipose tissue OR autologous micro-fragmented adipose tissue OR autologous microfragmented adipose tissue OR micro-fragmented lipoaspirate) | 71 |
| #7 | AB=(Autologous Microfragmented Fat Tissue OR Microfragmented Adipose Tissue OR Micro-fragmented adipose tissue OR autologous micro-fragmented adipose tissue OR autologous microfragmented adipose tissue OR micro-fragmented lipoaspirate) | 96 |
| #8 | #5 OR #6 OR #7 | 115 |
| #9 | #4 AND #8 | 62 |

*Search strategy of CNKI*

检索范围 (scope)：**总库**  (all databases)（主题 (TS)：骨关节炎 (osteoarthritis)（精确）(precise)） AND （（主题 (TS)：膝 (knee)（精确）(precise)）） AND （（主题 (TS)：自体 (autologous)（精确）(precise)））AND（主题 (TS)：脂肪 (fat or adipose)（精确） (precise)）） 3

*Search strategy of CQVIP*

题名或关键词 (title or keywords)=骨关节炎 (osteoarthritis) AND 题目或关键词 (title or keywords)=膝 (knee) AND 题名或关键词 (title or keywords)=自体 (autologous) AND 题名或关键词 (title or keywords)=脂肪 (fat or adipose) 3

*Search strategy of Wanfan DATA*

题名或关键词 (title or keywords): (骨关节炎 [osteoarthritis]) and 题名或关键词 (title or keywords): (膝 [knee]) and 题名或关键词 (title or keywords): (自体 [autologous]) and 题名或关键词 (title or keywords): (脂肪 [fat or adipose]) 11
