# Supplementary material for: Autologous micro-fragmented adipose tissue in the treatment of atherosclerosis patients with knee osteoarthritis in geriatric population: A systematic review and meta-analysis
Source: PLoS One. 2023 Aug 31;18(8):e0289610. doi: 10.1371/journal.pone.0289610 (PMC10470951; doi:10.1371/journal.pone.0289610)
Supplement: S2 Table — (DOCX) [file pone.0289610.s009.docx]

**Supplementary Table 2.** Quality assessment of the included studies

| Study (MINORS) | A clearly stated aim | Inclusion of consecutive patients | Prospective collection of data | Endpoints appropriate to the aim of the study | Unbiased assessment of the study endpoint | Follow-up period appropriate to the aim of the study | Loss to follow up less than 5% | Prospective calculation of the study size | Total |
| --- | --- | --- | --- | --- | --- | --- | --- | --- | --- |
| Boric, 2019 | 2 | 2 | 2 | 2 | 1 | 2 | 2 | 0 | 13 |
| Cattaneo, 2018 | 2 | 2 | 0 | 2 | 1 | 2 | 2 | 0 | 11 |
| Genechten, 2021 | 2 | 1 | 2 | 2 | 1 | 2 | 2 | 0 | 12 |
| Heidari, 2020 | 2 | 1 | 1 | 2 | 1 | 2 | 2 | 0 | 11 |
| Hudetz, 2019 | 2 | 1 | 2 | 2 | 1 | 2 | 2 | 0 | 12 |
| Malanga, 2020 | 2 | 1 | 2 | 2 | 1 | 2 | 2 | 0 | 12 |
| Mautner, 2019 | 2 | 1 | 0 | 2 | 1 | 2 | 2 | 0 | 10 |
| Russo, 2017 | 2 | 1 | 0 | 2 | 1 | 2 | 2 | 0 | 10 |
| Baria, 2022 | 2 | 2 | 2 | 2 | 1 | 2 | 1 | 0 | 12 |
| Gobbi, 2022 | 2 | 2 | 2 | 2 | 1 | 2 | 2 | 2 | 15 |
| Screpis, 2022 | 2 | 1 | 0 | 2 | 1 | 2 | 2 | 0 | 10 |
| Zaffagnin, 2022 | 2 | 2 | 2 | 2 | 2 | 2 | 1 | 2 | 15 |
| Liang, 2018 | 2 | 2 | 2 | 2 | 1 | 0 | 2 | 0 | 11 |
| Li, 2017 | 2 | 2 | 2 | 2 | 1 | 2 | 2 | 0 | 13 |
| Jonsson, 2011 | 2 | 1 | 0 | 2 | 1 | 2 | 2 | 0 | 10 |
| Hoeven, 2013 | 2 | 1 | 0 | 2 | 1 | 2 | 2 | 0 | 10 |
| Hoeven, 2015 | 2 | 1 | 0 | 2 | 1 | 2 | 2 | 0 | 10 |
| Gielis, 2017 | 2 | 1 | 2 | 2 | 1 | 2 | 2 | 0 | 12 |
| Hussain, 2015 | 2 | 1 | 2 | 2 | 1 | 2 | 2 | 0 | 12 |
