# Supplementary material for: Autologous micro-fragmented adipose tissue in the treatment of atherosclerosis patients with knee osteoarthritis in geriatric population: A systematic review and meta-analysis
Source: PLoS One. 2023 Aug 31;18(8):e0289610. doi: 10.1371/journal.pone.0289610 (PMC10470951; doi:10.1371/journal.pone.0289610)
Supplement: Attachment — Submitted filename: reviewers comment to Author.docx [file pone.0289610.s010.docx]

The study is interest. It is a meta-analysis study of Autologous micro-fragmented adipose tissue in the treatment of atherosclerosis patients with knee osteoarthritis in the elderly population. It needs Major Revision by a native English speaker.

The abstract section is lengthy and needs recast. The Background should be brief, describing the rational for the study. The purpose of the study itemized in this section should be removed completely, rather, summarize the purpose briefly in a prose form. The acronyms should be removed. The methods poorly written.

Your choice of words “We can speculate that it has a 30 comorbid relationship based on its pathogenesis and common risk factors” seems worrisome. In research of this nature assumptions should be avoided

Results looks misleading. Let the comparison reflect only variables that have significant correlation. The values represent what? Is it Pre and Post treatment.

Please let your conclusion reflect your findings and should be based on the focus of your study

Your Literature review section is extremely short. I recommend you do a thorough review of this section and discuss exclusively the variables in the study, focusing on previous studies in line with Autologous micro-fragmented adipose tissue in the treatment of atherosclerosis and its prevalence in Geriatric Population

The interpretation of results is confusing. You had a curious pre- and post-treatment result not reflecting micro-fragmented adipose tissue in the treatment of atherosclerosis patients with knee osteoarthritis for the 14 included selected study. I believe it is of utmost importance that the authors confront and compare both results in the discussion, raising points regarding the therapeutic efficacy and safety of autologous MFAT for the treatment of knee OA.

I feel that you could be more specific concerning the interaction between your discussion which eventual will form the basis of your conclusion

Your recommendations in line 346 – 348 reveals no connection from your conclusion.

Your list of references fails to tally with your in-text references. It is questionable and needs careful review.

Thank you for submission.
